# North African Influences and Potential Bias in Case-Control Association Studies in the Spanish Population

**DOI:** 10.1371/journal.pone.0018389

**Published:** 2011-03-30

**Authors:** María Pino-Yanes, Almudena Corrales, Santiago Basaldúa, Alexis Hernández, Luisa Guerra, Jesús Villar, Carlos Flores

**Affiliations:** 1 CIBER de Enfermedades Respiratorias, Instituto de Salud Carlos III, Madrid, Spain; 2 Research Unit, Hospital Universitario N.S. de Candelaria, Tenerife, Spain; 3 Instituto Nacional de Toxicología y Ciencias Forenses, Delegación de Canarias, Tenerife, Spain; 4 Hematology Service, Hospital Universitario Dr. Negrín, Las Palmas de Gran Canaria, Spain; 5 Multidisciplinary Organ Dysfunction Evaluation Research Network, Research Unit, Hospital Universitario Dr. Negrín, Las Palmas de Gran Canaria, Spain; 6 Keenan Research Center, St. Michael's Hospital, Toronto, Canada; University of Utah, United States of America

## Abstract

**Background:**

Despite the limited genetic heterogeneity of Spanish populations, substantial evidences support that historical African influences have not affected them uniformly. Accounting for such population differences might be essential to reduce spurious results in association studies of genetic factors with disease. Using ancestry informative markers (AIMs), we aimed to measure the African influences in Spanish populations and to explore whether these might introduce statistical bias in population-based association studies.

**Methodology/Principal Findings:**

We genotyped 93 AIMs in Spanish (from the Canary Islands and the Iberian Peninsula) and Northwest Africans, and conducted population and individual-based clustering analyses along with reference data from the HapMap, HGDP-CEPH, and other sources. We found significant differences for the Northwest African influence among Spanish populations from as low as ≈5% in Spanish from the Iberian Peninsula to as much as ≈17% in Canary Islanders, whereas the sub-Saharan African influence was negligible. Strikingly, the Northwest African ancestry showed a wide inter-individual variation in Canary Islanders ranging from 0% to 96%, reflecting the violent way the Islands were conquered and colonized by the Spanish in the XV century. As a consequence, a comparison of allele frequencies between Spanish samples from the Iberian Peninsula and the Canary Islands evidenced an excess of markers with significant differences. However, the inflation of *p*-values for the differences was adequately controlled by correcting for genetic ancestry estimates derived from a reduced number of AIMs.

**Conclusions/Significance:**

Although the African influences estimated might be biased due to marker ascertainment, these results confirm that Northwest African genetic footprints are recognizable nowadays in the Spanish populations, particularly in Canary Islanders, and that the uneven African influences existing in these populations might increase the risk for false positives in association studies. Adjusting for population stratification assessed with a few dozen AIMs would be sufficient to control this effect.

## Introduction

Populations inhabiting the Iberian Peninsula have been influenced by the same major human prehistoric migrations that have affected the rest of European populations, regardless of the extent to which Neolithic expansions from the Near East have influenced their genetic makeup [1]–[5]. Y-chromosome studies have indicated that such migrations have influenced uniformly the Iberian genetic background [6]. Thus, with the exception of a few isolates, the Basques being the best characterized representatives [7], populations inhabiting the Iberian Peninsula show a substantial genetic homogeneity [1], [6], [8], [9].

Despite this, several lines of evidence support the existence of identifiable unequal African influences in populations from Spain, both from the Iberian Peninsula and the Canary Islands. The excess of diversity observed in Southwestern Europeans for genome-wide autosomal haplotypes was interpreted as resulting from direct migrations from North Africa across the Mediterranean [10]. In addition, mitochondrial DNA (mtDNA) and Y-chromosome studies have revealed geographically clustered North African influences of about 8–10% in Iberia [6], [8], [11]–[13], with somewhat higher estimates for populations of the Northwestern and Southern regions [6], [8], [12], [14] that have been typically reconciled with a main historical migration from the nearby Northwest Africa as part of the Islamic rule starting in 711 CE and lasting seven centuries [15]. Besides, genetic footprints of important African influences have been demonstrated for particular Spanish populations such as the Canary Islanders, for which many studies have suggested a genetic influence of 22–38% from Northwest Africans, and less than 5% from sub-Saharan Africans [16]–[21]. The historical admixture of Spanish colonizers with aborigines related to Northwest African Berbers, and with sub-Saharan Africans introduced as a result of slave trade, has been postulated as an explanation to account for the peculiarities of this population [17].

These major documented historical influences from divergent populations such as those from the nearby North Africa [22], [23] are likely to have introduced subtle population differences among Spanish that might be considered in genetic epidemiology studies [13]. Accounting for such genetic differences (i.e. population stratification) is imperative to reduce false positive or negative results in case-control and cohort studies of association of genetic variants with disease [24], [25]. In addition, a better characterization of the different genetic strata that are present in the population would enable future studies to address if genetic factors might underlie disparities among Spanish populations for the incidence of complex illness such as asthma [26], type 2 diabetes [27], and hypertension [28].

Ancestry informative markers (AIMs), i.e. genetic loci showing large allele frequency differences between populations, allow accurate apportioning of genetic influences in populations [29] and are useful to efficiently account for population stratification in genetic epidemiology studies with unrelated individuals where dense genotype data is not available [30]. In studies where samples from different European populations are compared, population stratification effects can be controlled by using a few hundred autosomal AIMs, termed EuroAIMs [31], which recover the largest features of the north-northwest (NNW) to south-southeast (SSE) axis of genetic differentiation in Europe [30], [31]. A set of AIMs ascertained to specifically distinguish North African and European ancestries is not available in the literature. However, given that AIMs tend to be informative for ancestry inference within several world regions [29], we reasoned that previously selected EuroAIMs were likely to be informative for ancestry as well in these populations. Based on this principle, here we first assessed the ability of EuroAIMs to distinguish Spanish, Northwest and sub-Saharan Africans, and next utilized them to identify African influences in Spanish populations. We finally compared allele frequencies between Spanish to illustrate the potential effects of including samples with uneven African influences in population-based association studies.

## Results

The potential ancestry informativeness of EuroAIMs for African and Spanish populations was initially evidenced by comparing genome-wide data from reference samples from the Human Genome Diversity Panel (HGDP) [32] (see explanations in Text S1). Subsequently, samples from outbred populations from Northwest Africa and Spain (from the Iberian Peninsula and the Canary Islands) were genotyped for 93 EuroAIMs. In the following, samples and populations from any part of Spain, either from the Iberian Peninsula or from the Canary Islands, will be referred to as Spanish. We reserved the term Iberian to refer to samples and populations from the Iberian Peninsula. Out of the 93 EuroAIMs, seven markers departed significantly from Hardy-Weinberg equilibrium (HWE) in at least one of these populations (Table S1). However, after considering the multiple comparisons using a Bonferroni-like correction (significance at *p*-value = 0.00054), only two markers departed significantly from HWE: rs1073321 in Canary Islanders and rs7277342 in Northwest Africans. Given that not a single marker deviated from HWE after this correction in all three genotyped samples, and that genotyping was performed simultaneously for all samples obtaining similarly high completion rates (≥97%), we interpreted that HWE departures were more likely to be related to chance and retained all 93 EuroAIMs for further studies. On average, genetic differentiation levels (F_ST_) obtained for the comparison of the 93 EuroAIMs between Northwest Africans and Iberians, Northwest Africans and Yoruba Nigerians (YRI), and Iberians and YRI were 0.0422, 0.100 and 0.255, respectively (further information in Table S1). The three estimates were slightly lower when Canary Islanders were considered for comparisons instead of Iberians. These differentiation levels strongly support that there is enough information on the EuroAIMs set to dissect the sub-Saharan Africans from Iberians and Northwest Africans, and also to distinguish the latter two populations.

### Population and individual clustering using EuroAIMs

**Figure 1:**
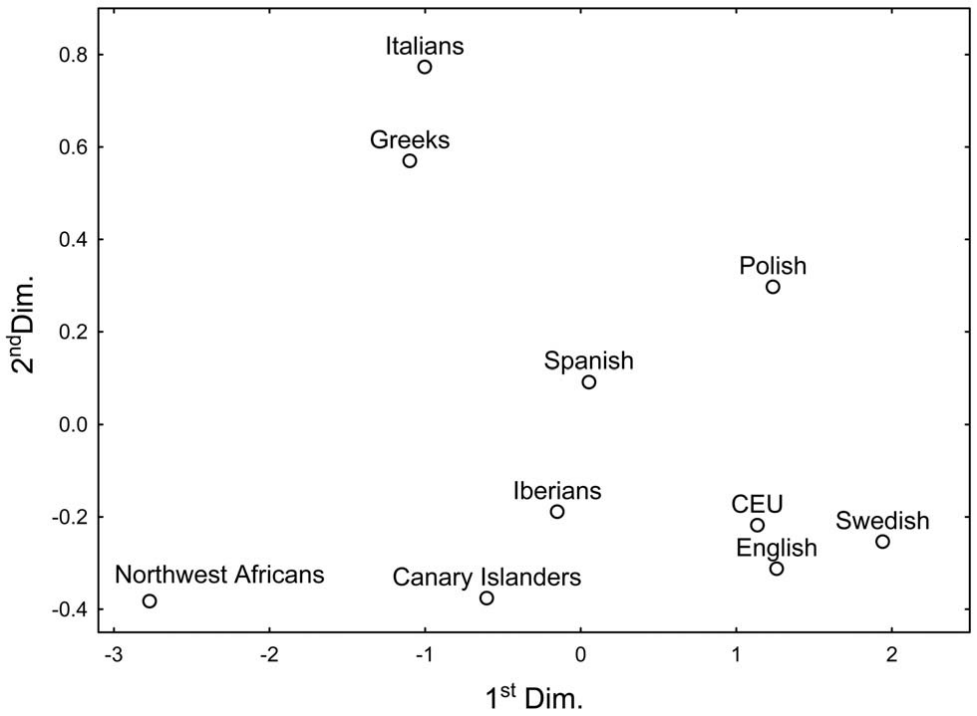
F_ST_-based multidimensional scaling plot. The *r* ^2^ of F_ST_ distances to plot-derived distances was 0.995. The stress value was 0.036, indicating that additional dimensions were not necessary. Spanish, samples from Price et al. [30]; CEU, Utah residents with ancestry from northern and western Europe from HapMap [43].

**Figure 2:**
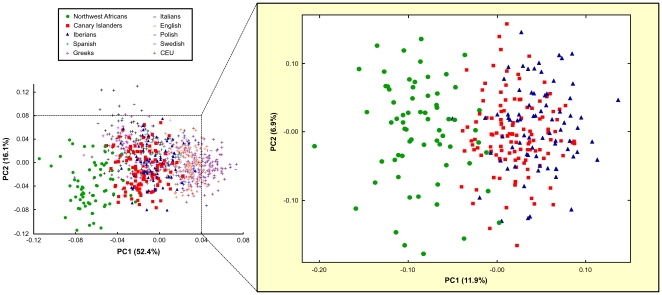
Plot of the top two principal components from the analysis of populations. Results from the analysis based on the 93 EuroAIMs restricted to Iberians, Northwest Africans and Canary Islanders are represented on the right panel. The percentage of explained variation is indicated in each axis. Spanish, samples from Price et al. [30]; CEU, Utah residents with ancestry from northern and western Europe from HapMap [43].

To explore to which extent population groups were separated from each other, population and individual-based analyses were performed. For these analyses, sub-Saharan African populations were not included due to their considerable divergence to the rest of populations. Multidimensional scaling analysis of pairwise population F_ST_ genetic distances revealed three well separated clusters of populations (Figure 1): a clear separation of Northwest Africans from European populations, and of Southern from Northern European populations in agreement with previous observations [30], [33]. Principal component analysis (PCA) of individuals revealed a similar clustering pattern (Figure 2) with two significant principal components (PCs) accounting for 68.5% of variance and clearly separating Northwest Africans from Europeans: PC1 distinguishing Northwest Africans from NNW Europeans (*p* = 6.23E-17) and PC2 differentiating Northwest Africans from SSE Europeans (*p* = 3.88E-17). Spanish populations were assigned at intermediate positions in the NNW-SSE axis of European differentiation, with Canary Islanders clustering in their vicinity, albeit showing a slight shift towards Northwest Africans (Figure 2). As a support for the admixed origin of Canary Islanders, restricting the PCA to Iberians, Northwest Africans and Canary Islanders only revealed one significant axis of variation separating Iberians from Northwest Africans (*p* = 8.93E-17) (Figure 2), as would be expected for a typical admixed population because of the linear mixing of allele frequencies in the parental populations [34].

Among Spanish, the F_ST_ distances of Canary Islanders to Iberians and to a Spanish sample without information of sampling location [30] were at least four times larger than the distance between the latter samples (Table S2). Notably, small F_ST_ distances were obtained for different partitions of the Iberian sample (not shown), including a separation on an East-West axis as noted in a previous study [12]. Thus, according to these markers, Spanish populations show a reduced genetic heterogeneity, with Canary Islanders constituting the main source of population stratification among the samples analyzed. Although these markers were ascertained to recover the largest features of the NNW-SSE axis of genetic differentiation in Europe and not dissect the genetic heterogeneity of Spanish populations, these results are in agreement with results from previous studies using alternative marker sets [9].

To verify that population relationships were not affected by selection processes, we re-assessed population and individual-based analyses excluding the EuroAIMs with potential effects on gene function according to the SNP Function Portal [35] (i.e. predicting coding non-synonymous changes, disrupting predicted miRNA target sites or being located within gene UTRs that can affect mRNA stability) (Table S3). The results with or without the EuroAIMs with predicted function were largely similar, albeit the distinction of Northwest Africans from Southern European populations was not as apparent when these markers were excluded from the analyses (not shown). Thus, given that we aimed to accurate apportion the African influences in Spanish populations, we retained all 93 EuroAIMs for further studies.

Taken together, these observations support the genetic discontinuity observed between Northwest Africans and their closest European neighbors [23], [36], and the usefulness of previously ascertained EuroAIMs to recover the genetic ancestry of Northwest Africans and Europeans.

### African influences in Spanish populations

As there is not a formal way to recognize if minor individual ancestries are accurate measurements or artifacts of the methods used, we first assessed the influences at population level with the only purpose of deciding which populations to include in STRUCTURE analyses. For that, we evaluated African influences in Canary Islanders as, among Spanish populations, it would be more likely to detect such influences in this population based on the historical records. Given that results from a three-population model of genetic contributions in Canary Islanders indicated that a direct sub-Saharan African influence in this population was negligible (Table 1), and that sub-Saharan Africans were clearly differentiated from the other two parental populations (Iberians and Northwest Africans) by means of an unsupervised assignment of individuals to populations (Figure S1), we excluded sub-Saharan Africans from further analyses. The Northwest African influence in Canary Islanders was estimated in ≈23% (Table 1), which was in the range of previous estimates [17], [21]. Nevertheless, it is likely that this estimate is biased upwards. Note that a portion of the Northwest African component in Canary Islanders might be due to the Northwest African influences in the Iberian colonizers, given the gene flow between Northwest Africans and Iberians (see results below).

**Table 1:** Population-based estimates (95% confidence interval) of genetic contributions in Canary Islanders using 93 EuroAIMs.

Model	Estimator^a^	Iberian	Northwest African	Sub-Saharan African
3-populations	m_R_	0.733 (0.666, 0.809)	0.294 (0.190, 0.391)	−0.027 (−0.064, 0.012)
3-populations	m_W_	0.765 (-, -)^b^	0.234 (-, -)^b^	3.8E-5 (-, -)^b^
2-populations	m_R_	0.751 (0.696, 0.818)	0.249 (0.182, 0.304)	-
2-populations	m_W_	0.772 (0.564, 0.898)	0.228 (0.102, 0.436)	-

a m_R_: Moment estimator [47], m_W_: Maximum-likelihood estimator [46];

b Computational burden precluded the adequate estimation of boundaries when the number of parental populations was set to three.

**Figure 3:**
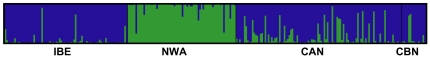
STRUCTURE results based on EuroAIMs. Each vertical line represents an individual where colors indicate the proportion of the individual's genome derived from each of the two inferred populations. CAN, Canary Islanders collected for this study; CBN, Canary Islanders available from the National Spanish DNA Bank; IBE, Iberians; NWA, Northwest Africans.

After excluding sub-Saharan Africans from the study, STRUCTURE identified two populations corresponding to Northwest Africans and Spanish (Figure 3). With few exceptions, which may represent unreported recent admixture, Northwest African and Iberian samples showed overwhelming contribution from a single population. Similar results were observed using subsets of as few as 23 of the markers ranking higher for different measures of ancestry informativeness, albeit at the cost of as much as 11% of individuals being classified in the incorrect population (see Text S2 for further details). Strikingly, the Northwest African influence in Canary Islanders was 17.4% on average (within the 95% confidence interval of population-based methods) while it was significantly lower in Iberians (≈5%; two-tailed t-test *p* = 1.00E-6) (Table 2). In addition, a large inter-individual variation was observed for the Northwest African ancestry among Canary Islanders (range 0.0–95.7%). Surprisingly, about 9% of Canary Islanders showed ≥70% of Northwest African ancestry. The average Northwest African ancestry was neither different between the Canary Islanders collected for this study and those from the National Spanish DNA Bank (two-tailed t-test *p* = 0.488), nor among the samples from the different islands (ANOVA *p* = 0.213) (Table 2). Note, however, that estimates by island must be interpreted with caution given the small sample sizes. Comparable results were obtained with subsets of as few as 69 of the markers ranking higher for different measures of ancestry informativeness (Text S2). With fewer markers, the estimated Northwest African influences in Canary Islanders resulted severely biased upwards as a consequence of the reduction of information.

**Table 2:** Average Northwest African ancestry estimates.

Population	Mean (SD)
Iberians (n = 77)	0.050 (0.148)
Northwest Africans (n = 68)	0.947 (0.148)
Canary Islanders, CBN^a^ (n = 15)	0.126 (0.252)
Canary Islanders, CAN^b^ (n = 104)	0.174 (0.253)
Canary Islanders^b^, by island:	
El Hierro (n = 7)	0.198 (0.306)
La Palma (n = 7)	0.210 (0.330)
La Gomera (n = 7)	0.425 (0.389)
Tenerife (n = 30)	0.143 (0.253)
Gran Canaria (n = 30)	0.124 (0.181)
Lanzarote (n = 13)	0.164 (0.189)
Fuerteventura (n = 10)	0.216 (0.267)

a Samples from the National Spanish DNA Bank;

b Samples collected for this study.

### Controlling population stratification in population-based association studies in Spanish populations using EuroAIMs

To limit population stratification effects in a typical case-control association study with unrelated individuals, samples are usually matched by political boundaries [37]. Because of the estimated disparities in Northwest African influences among the Spanish populations, we expected that multicentric studies, with countrywide sampling schemes, would be at risk of an increased rate of false positives. As a proof of principle, a comparison of the allele frequency differences of the 93 EuroAIMs between Iberians and Canary Islanders using the Cochran-Armitage trend test resulted in 12 tests with *p*<0.05 (Table 3). At 5% type-I error rate, only 4.65 markers were expected to be significant by chance, indicating the existence of an excess of markers with large differences between both samples (χ^2^ test *p* = 0.0375). To explore if this effect was controlled by using the ancestry information derived from EuroAIMs, we adjusted these comparisons using logistic regression models including as covariates either the STRUCTURE Northwest African individual ancestries or the PCA scores. Adjusting for STRUCTURE estimates, based on the full marker set or any of the subsets of markers ranking higher for different measures of ancestry informativeness, barely decreased the number of significant tests (Table 3), while significance levels dropped considerably (Figure S2). As an example, the smallest *p*-value, which was obtained for the marker with the largest F_ST_ between Iberians and Northwest Africans (0.4153), increased from *p* = 0.0002 with the unadjusted test (the Cochran-Armitage trend test) to *p* = 0.006 after the adjustment. The first *p*-value will remain significant after a Bonferroni-like correction for the multiple comparisons, but the second will not. Nevertheless, the adjustment of comparisons using the PC1 scores not only resulted in reduced significance levels but also in an effective control of false positives at 5% rate (Table 3; Figure S2). Results did not improve if the scores from the PC2 were also used for the adjustments (not shown).

**Table 3:** Fraction of markers with significant differences between Canary Islanders and Iberians.

Comparison	Markers with *p*≤0.05
Unadjusted Cochran-Armitage trend test	0.29
Adjusted by STRUCTURE estimates:	
93	0.118
69	0.108
46	0.097
23	0.086
Adjusted by PCA^a^ scores:	
93	0.097
69	0.053
46	0.065
23	0.065

a Using PC1 from the PCA restricted to Iberians, Northwest Africans and Canary Islanders.

## Discussion

We have illustrated that a few dozen EuroAIMs ascertained to distinguish the NNW-SSE axis of genetic differentiation in Europe could also be utilized to distinguish Northwest Africans from European populations. Their use allowed us to accurately distinguish that Northwest African influences were minor in populations from the Iberian Peninsula, while these were substantially greater in Canary Islanders, ranging from as low as 0% to as high as 96% among individuals. We finally demonstrated that these African influences might constitute a potential source of population stratification in population-based association studies conducted in Spanish populations, and that this effect was appropriately controlled using a reduced number of EuroAIMs.

It was not unexpected to find a low level of bidirectional gene flow (5%) between Northwest Africans and Iberians given the results from previous studies [22], [23]. However, while the North African influence in Iberian populations has been estimated in 8–10% based on markers with uniparental inheritance [6], [12], [13], we found slightly lower levels using autosomal markers, ranging from as low as 2.1% (SD = 4.6) in Eastern Iberians to as much as 9.0% (SD = 23.8) in Western Iberian samples, although these differences did not reach statistical significance (two-tailed t-test *p* = 0.156). Strikingly, a parallel geographical clustering of the Northwest African influence in Iberian populations was recently revealed with Y-chromosome binary markers [12]. Given that in this study, Iberians were represented by a small number of samples from different mainland localities, it remains interesting to explore if such geographical patterning of the Northwest African influence is confirmed in reasonably-sized population samples from different localities.

The substantial Northwest African ancestry found for Canary Islanders supports that, despite the aggressive conquest by the Spanish in the XV century and the subsequent immigration, genetic footprints of the first settlers of the Canary Islands persist in the current inhabitants. Paralleling mtDNA findings [16], the largest average Northwest African contribution was found for the samples from La Gomera. Remarkably, the sub-Saharan African influence was unnoticeably in this study, despite our results support that the EuroAIMs set contains enough information to distinguish sub-Saharan Africans from the other populations analyzed. This result contrasts with the previous evidences provided by mtDNA [16] and Y-chromosome [18], and the documented early introduction of sub-Saharan Africans after the conquest as a result of slave trade [17]. However, given the differences in inheritance among these loci, and the complex history of Canary Islanders [17], it is not surprising to reach different but complementary conclusions examining different loci [17], [21]. On this basis, we found little support for a sub-Saharan African influence on Canary Islanders other than the introduced through the first settlers, given that genetic studies in aboriginal remains demonstrate the existence of a Saharan substrate [21] and that modern Western Saharan populations show substantial sub-Saharan African influences [22], [23].

In view of the disparate estimates of the African influences obtained for the two Spanish populations analyzed, we anticipated an increase of false positive results in population-based association studies of genes with disease simply due to systematic differences in such influences. Using PC scores and, alternatively, individual admixtures obtained by means of STRUCTURE, we have shown that there is a potential benefit of correcting for population stratification in Spanish studies including samples from Canary Islanders. If no adjustment was done, the number of false positive associations resulted in more than twice the expected amount under the null hypothesis. Thus, even small levels of population admixture can undermine an association study and lead to false positive results [37]. On the contrary, at an affordable cost, genotyping a few dozen EuroAIMs would allow adjusting the association tests for population stratification to control the excess of spurious results. This does not imply that African influences are the only source of population stratification in Spanish. Previous studies have indicated that even if both cases and controls are collected from the same European country [38] or when comparing samples across European populations, it might be necessary to control for population stratification [33]. Although this study must be viewed simply as an exercise under particular worst sample settings, our results suggest that, at least, those single nucleotide polymorphisms (SNPs) with large allele frequency differences among Iberians and Northwest Africans might be at risk of being detected as false positives in association studies conducted in Spanish populations. Given the large sample sizes needed to detect the association of genetic variants with modest effects in disease [25], the statistical bias introduced may become more pronounced in real settings [39].

We warn that because we used markers showing large allele frequency differences between populations [30], the reported genetic differences between Spanish and Northwest African populations suffer from ascertainment bias, therefore, not corresponding to the average of the genome. This study also shares the limitations of any other genetic study aiming to estimate ancestry proportions. Among others, ancestry proportions are highly dependent on the samples considered as references or parentals, which are usually derived from a reduced number of individuals from contemporary populations. In this sense, we used a mixture of samples from different locations in Morocco to quantify North African influences in Iberians and as a proxy for the aboriginal population inhabiting the Canary Islands before the conquest. This is a reasonable assumption, given the evidence from the historical records [15], [17] and the numerous previous genetic studies [12], [16]–[19], [21], [40]. Besides, we admit that the use of this sample to represent the North African population constitutes a simplification of the heterogeneous source of African influences that have affected the Spanish populations. It is likely that further sampling of North African regions and the typing of additional markers might allow identifying other influences in Spanish, as has been illustrated recently for African Americans [41].

In conclusion, we have extended the use of EuroAIMs to allow distinguishing the contrasting Northwest African influences existing among the Spanish populations. We have also demonstrated that these differences might increase the risk for false positives in genetic epidemiology studies that can be effectively controlled using a reduced number of EuroAIMs.

## Materials and Methods

### Ethics statement

This study was approved by the Hospital Universitario N.S. de Candelaria and Hospital Universitario Dr. Negrín Ethics Committees. Written informed consent was obtained from all participants involved in the study.

### Samples

DNA samples from individuals of the general Iberian population were obtained from the Spanish National DNA Bank (www.bancoadn.org), consisting on 77 samples assigned to different Iberian localities based on the individual's self-reported grandparental origin: Andalusia (n = 15), Murcia (n = 5), Extremadura (n = 5), Castile-La Mancha (n = 4), Valencia (n = 7), Castile and Leon (n = 9), Madrid (n = 1), Catalonia (n = 2), Galicia (n = 4), Cantabria (n = 3), Navarre (n = 4), La Rioja (n = 4), Asturias (n = 1), and mixed Iberian origin (n = 13). The Canary Island population was represented by DNA samples from 104 unrelated healthy donors with at least two generations of ancestors born in the Canary Islands collected for the study (El Hierro, n = 7; La Palma, n = 7; La Gomera, n = 7; Tenerife, n = 30; Gran Canaria, n = 30; Lanzarote, n = 13; Fuerteventura, n = 10), and 15 additional samples from Gran Canaria obtained from the Spanish National DNA Bank. Additionally, 68 DNA samples from unrelated healthy individuals with at least two generations of Northwest African descent were studied: 25 from Casablanca, 25 from Rabat, and 18 from other regions of Morocco. Details of laboratory procedures can be found in the Text S3.

### Reference population data

To get an initial evidence of ancestry informativeness of EuroAIMs for African and Spanish ancestries, genome-wide data from 29 Mozabite Algerians and 24 French Basques from the HGDP [32] were used as representatives for North African and Spanish populations, although both are well-known population isolates [7], [42]. Genotypes from 60 unrelated YRI [43] were used to represent the sub-Saharan African population. To place the study in a European context, we utilized previously published data from 163 Swedish, 57 Polish, 76 English, 119 Italians, 68 Greeks, and 55 Spanish [30], as well as from 60 Utah residents with ancestry from northern and western Europe (CEU) [43].

### Genotyping

Samples were genotyped for EuroAIMs recovering the largest features of the European NNW-SSE axis of differentiation [30], focusing on those 93 SNPs that overlapped across all European samples used in the original study (Table S1). Genotyping was conducted utilizing the iPLEX™ Gold assay on MassARRAY system (Sequenom, San Diego, CA) by the Spanish National Genotyping Center, Santiago de Compostela Node (CeGen, http://www.cegen.org). Nine SNPs that gave poor quality data on this platform were finally determined using SNaPshot® Multiplex Kit reactions (Applied Biosystems, Foster City, CA) (Text S3). Genotypes are available from the corresponding author upon request.

### Statistical analysis

#### Summary statistics

Allele counts, exact tests for HWE departures [44], and relevant measures of ancestry informativeness of EuroAIMs were calculated using the SNPInfostats software (available from the corresponding author upon request). For each marker, SNPInfostats was used to estimate the absolute allele frequency difference (δ), the Weir & Cockerham F_ST_ genetic distance [45], as well as the informativeness of assignment index *I*_n_ [29].

#### Population relationships

EIGENSOFT [24] was used to calculate pairwise population F_ST_ genetic distances, to assess PCA of individual samples, and to determine the ANOVA statistics for population differences along each of the PCs. Multidimensional scaling was used to represent pairwise population F_ST_ genetic distances in two dimensions using SPSS ver.15 (SPSS Inc., Chicago, IL).

#### Ancestry assessment

In order to obtain initial estimates of the genetic contribution of populations in Canary Islanders, particularly the influence of sub-Saharan Africans, population-based estimates were calculated using LEADMIX [46] by means of the m_R_ least-squares estimator, that ignores sampling and genetic drift in populations [47], and the m_W_ maximum likelihood estimator, that allows estimating genetic contributions taking into account the effects of sampling and genetic drift in all populations and the differentiation between parental populations before the admixture event [46].

Individual ancestry estimates were assessed using an unsupervised assignment of individuals to populations by means of STRUCTURE 2.2 [48]. To infer the number of populations (K), three independent runs with a burn-in length of 50,000 for 200,000 repetitions from K = 1 to K = 7 were performed, using a correlated allele frequency with no-admixture model (as suggested by the software documentation to detect subtle structure) and setting lambda at 1. K was deduced from all runs using the method of Evanno et al. [49], which is based on the rate of change in the estimate of the posterior probability of the data with respect to successive K values. Additional runs with longer iterations were also carried out to check the consistency of the results.

#### Ancestry inference with marker subsets

With the purpose of exploring the ability of reduced EuroAIMs subsets to estimate population membership and ancestry proportions, we selected three subsets containing the most informative 23, 46 and 69 markers. For that, EuroAIMs were ranked based on the average of ranks for *I*_n_ and δ values, given that F_ST_ and *I*_n_ values were highly correlated (*r* ^2^ = 0.99). STRUCTURE was run for each subset using the same parameters as described above and all correctly identified K = 2. A cut-off membership of 0.70 was used as a criterion for the individual inclusion in the self-reported population. Pearson's correlations between ancestry estimates derived from each subset and those from the 93 EuroAIMs set were computed.

#### Allele frequency differences between Canary Islanders and Iberians

To explore whether allele frequencies of the EuroAIMs differed significantly among the Spanish samples, we treated each individual EuroAIM marker as a candidate locus for association in a mock study where Canary Islanders were taken as “cases” and Iberians were considered “controls”. Differences were first tested using the Cochran-Armitage trend test, which is similar to the allele count-test albeit not relying on the assumption of HWE [50]. In order to investigate whether there was a statistical benefit of correcting for population stratification, we then tested the differences adjusting the comparisons for population stratification by means of logistic regression models. These included the mock disease status as the dependent variable and the EuroAIM marker genotypes as well as the PC scores obtained from EIGENSOFT as independent covariates. For this purpose, the genotypes were re-coded with pre-specified scores of 0, 1 and 2 as disease risk probabilities based on the number of risk alleles in the genotypes. Alternatively, instead of PC scores, logistic regressions were assessed with the Northwest African ancestry estimates obtained from STRUCTURE as one of the independent covariates. Theses adjustments were repeated with PC scores and the Northwest African ancestry estimates obtained from the full marker set and subsets of the markers ranking higher for ancestry informativeness. All regression models were done by means of SNPassoc [51].

## Supporting Information

Figure S1 STRUCTURE results based on EuroAIMs. This analysis used data from Iberians (IBE), Northwest Africans (NWA) and Yoruba Nigerians (YRI) from HapMap [43] without using any prior population assignment. The model with best likelihood was K = 3 subpopulations. Each vertical line represents an individual where colors indicate the proportion of the individual's genome derived from each of the two inferred populations.
(TIF)

Figure S2 Quantile-quantile plots of *p*-values (as −log_10_P) for marker allele frequency differences between Spanish populations. Upper panel: adjustments based on STRUCTURE estimates; Lower panel: adjustments based on PC1 scores. Closed circles: trend test statistics; Open circles: statistics adjusted for estimates based on 93 EuroAIMs; dark grey circles: statistics adjusted for estimates based on 69 markers; triangles: statistics adjusted for estimates based on 46 markers; light grey circles: statistics adjusted for estimates based on 23 markers. The discontinuous line indicates the null distribution.
(TIF)

Table S1 Summary statistics for EuroAIMs used in the study.
(DOC)

Table S2 Pairwise population F_ST_ genetic distances.
(DOC)

Table S3 Functional annotation of EuroAIMs within RefSeq genes.
(DOC)

Text S1 Ancestry informativeness of EuroAIMs in samples from HGDP.
(DOC)

Text S2 Ancestry informativeness of EuroAIMs subsets.
(PDF)

Text S3 Details of laboratory procedures.
(DOC)
