# Supplementary figures and images for: North African Influences and Potential Bias in Case-Control Association Studies in the Spanish Population
Source: PLoS One. 2011 Mar 30;6(3):e18389. doi: 10.1371/journal.pone.0018389 (PMC3068190; doi:10.1371/journal.pone.0018389)

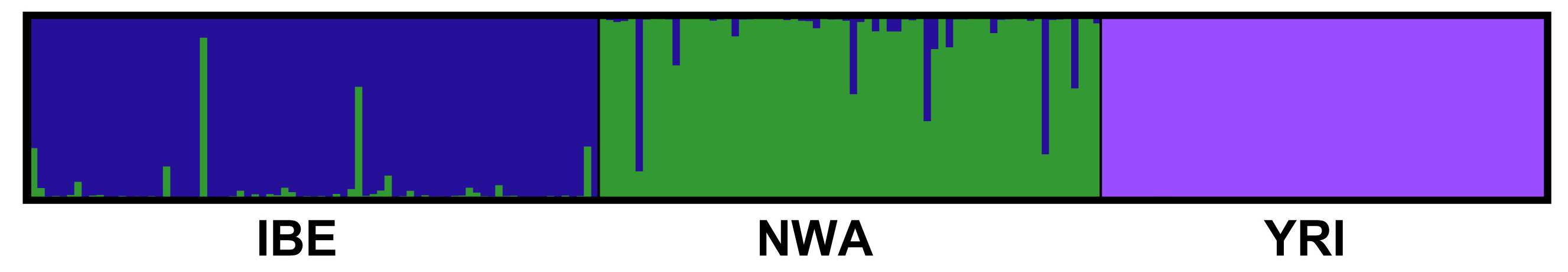

Supplement: Figure S1 — STRUCTURE results based on EuroAIMs. This analysis used data from Iberians (IBE), Northwest Africans (NWA) and Yoruba Nigerians (YRI) from HapMap [43] without using any prior population assignment. The model with best likelihood was K = 3 subpopulations. Each vertical line represents an individual where colors indicate the proportion of the individual's genome derived from each of the two inferred populations. (TIF) [file pone.0018389.s001.tif]

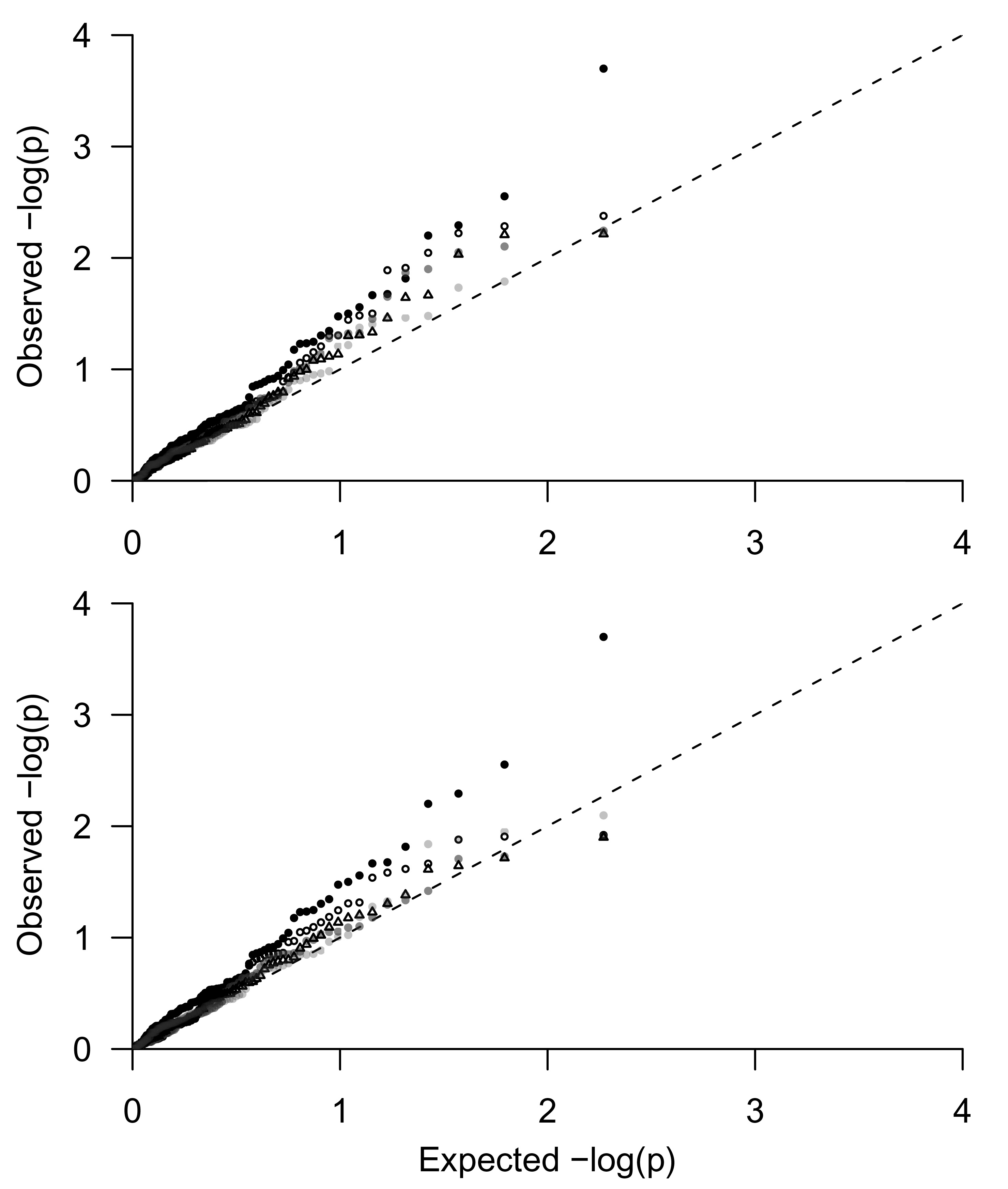

Supplement: Figure S2 — Quantile-quantile plots of p-values (as −log10P) for marker allele frequency differences between Spanish populations. Upper panel: adjustments based on STRUCTURE estimates; Lower panel: adjustments based on PC1 scores. Closed circles: trend test statistics; Open circles: statistics adjusted for estimates based on 93 EuroAIMs; dark grey circles: statistics adjusted for estimates based on 69 markers; triangles: statistics adjusted for estimates based on 46 markers; light grey circles: statistics adjusted for estimates based on 23 markers. The discontinuous line indicates the null distribution. (TIF) [file pone.0018389.s002.tif]
