# Supplementary material for: North African Influences and Potential Bias in Case-Control Association Studies in the Spanish Population
Source: PLoS One. 2011 Mar 30;6(3):e18389. doi: 10.1371/journal.pone.0018389 (PMC3068190; doi:10.1371/journal.pone.0018389)
Supplement: Table S1 — Summary statistics for EuroAIMs used in the study. (DOC) [file pone.0018389.s003.doc]

| **Table S1** Summary statistics for EuroAIMs used in the study. | | | | | | | | | | | | | | | | | | | | | | |
| --- | --- | --- | --- | --- | --- | --- | --- | --- | --- | --- | --- | --- | --- | --- | --- | --- | --- | --- | --- | --- | --- | --- |
|  |  |  |  |  |  | Ref. allele frequency | | | | Hardy-Weinberg equilibrium *p*-value^b^ | | | | IBE-NWA ancestry measures | | | IBE-YRI ancestry measures | | | NWA-YRI ancestry measures | | |
| rs# | Alleles | Chr. | Position^a^ | Call (%) | Ref. | CIs | IBE | NWA | YRI | CIs | IBE | NWA | YRI | *In* | δ | F_ST_ | *In* | δ | F_ST_ | *In* | δ | F_ST_ |
| rs1157492 | C/T | 1 | 211627299 | 98.5 | C | 0.40 | 0.40 | 0.25 | 0.45 | 1.73E-01 | 3.43E-01 | 3.30E-01 | 7.86E-01 | 1.26E-02 | 1.49E-01 | 3.52E-02 | 9.40E-04 | 4.29E-02 | 0.00E+00 | 2.04E-02 | 1.92E-01 | 6.31E-02 |
| rs11807062 | A/C | 1 | 3176394 | 99.6 | C | 0.20 | 0.16 | 0.38 | 0.75 | 2.42E-01 | 4.02E-01 | 1.18E-01 | 4.76E-01 | 2.94E-02 | 2.13E-01 | 9.80E-02 | 1.86E-01 | 5.88E-01 | 5.16E-01 | 7.34E-02 | 3.75E-01 | 2.35E-01 |
| rs1416467 | A/G | 1 | 80470749 | 99.6 | A | 0.51 | 0.51 | 0.35 | 0.25 | 9.96E-02 | 8.22E-01 | **1.76E-02** | 7.14E-01 | 1.21E-02 | 1.54E-01 | 3.35E-02 | 3.69E-02 | 2.61E-01 | 1.19E-01 | 6.92E-03 | 1.07E-01 | 1.06E-02 |
| rs1890131 | C/T | 1 | 164329131 | 100.0 | T | 0.24 | 0.32 | 0.09 | 0.03 | 1.29E-01 | 1.00E+00 | 1.00E+00 | 1.00E+00 | 4.29E-02 | 2.30E-01 | 1.35E-01 | 8.72E-02 | 2.93E-01 | 2.33E-01 | 9.89E-03 | 6.32E-02 | 2.02E-02 |
| rs2236876 | A/G | 1 | 169749143 | 100.0 | A | 0.35 | 0.32 | 0.43 | 0.40 | 6.87E-01 | 6.12E-01 | 4.59E-01 | 7.82E-01 | 5.54E-03 | 1.02E-01 | 8.19E-03 | 2.93E-03 | 7.35E-02 | 0.00E+00 | 4.14E-04 | 2.83E-02 | 0.00E+00 |
| rs2419063 | A/G | 1 | 186988492 | 99.6 | G | 0.12 | 0.05 | 0.18 | 0.30 | 6.63E-01 | 1.00E+00 | 4.40E-01 | 1.00E+00 | 2.20E-02 | 1.32E-01 | 6.98E-02 | 5.77E-02 | 2.48E-01 | 1.97E-01 | 9.27E-03 | 1.16E-01 | 2.07E-02 |
| rs495347 | C/T | 1 | 17909228 | 97.8 | C | 0.35 | 0.27 | 0.48 | 0.65 | 2.22E-01 | 1.00E+00 | 6.28E-01 | 1.40E-01 | 2.26E-02 | 2.05E-01 | 7.34E-02 | 7.17E-02 | 3.73E-01 | 2.36E-01 | 1.44E-02 | 1.68E-01 | 3.95E-02 |
| rs725974 | G/T | 1 | 167344909 | 93.0 | G | 0.22 | 0.18 | 0.43 | 0.23 | 7.75E-01 | 4.42E-01 | 4.52E-01 | 1.00E+00 | 3.50E-02 | 2.41E-01 | 1.18E-01 | 1.83E-03 | 4.91E-02 | 0.00E+00 | 2.11E-02 | 1.92E-01 | 6.47E-02 |
| rs7552548 | A/G | 1 | 82775867 | 99.6 | G | 0.36 | 0.27 | 0.39 | 0.49 | 1.00E+00 | 1.51E-01 | 7.99E-01 | 1.20E-01 | 8.17E-03 | 1.20E-01 | 1.85E-02 | 2.64E-02 | 2.22E-01 | 8.69E-02 | 5.28E-03 | 1.02E-01 | 5.30E-03 |
| rs10496610 | C/G | 2 | 123757682 | 99.6 | G | 0.22 | 0.19 | 0.07 | 0.00 | 7.93E-01 | 1.00E+00 | **2.00E-02** | 1.00E+00 | 1.89E-02 | 1.29E-01 | 5.56E-02 | 7.28E-02 | 1.95E-01 | 1.65E-01 | 2.35E-02 | 6.62E-02 | 4.76E-02 |
| rs1364394 | C/T | 2 | 95617604 | 98.9 | T | 0.27 | 0.29 | 0.16 | 0.05 | 4.91E-01 | 1.00E+00 | 6.52E-01 | 1.00E+00 | 1.35E-02 | 1.37E-01 | 3.79E-02 | 5.37E-02 | 2.39E-01 | 1.55E-01 | 1.43E-02 | 1.02E-01 | 3.57E-02 |
| rs1448314 | A/G | 2 | 223811705 | 99.6 | A | 0.10 | 0.08 | 0.20 | 0.25 | 1.00E+00 | 1.00E+00 | 2.76E-01 | 7.16E-01 | 1.57E-02 | 1.21E-01 | 4.74E-02 | 2.82E-02 | 1.72E-01 | 9.60E-02 | 1.91E-03 | 5.15E-02 | 0.00E+00 |
| rs1517407 | A/G | 2 | 63401982 | 99.3 | G | 0.45 | 0.47 | 0.36 | 0.11 | 4.61E-01 | 3.55E-01 | 2.92E-01 | 1.00E+00 | 5.89E-03 | 1.07E-01 | 9.40E-03 | 8.26E-02 | 3.58E-01 | 2.45E-01 | 4.58E-02 | 2.51E-01 | 1.42E-01 |
| rs3769005 | C/G | 2 | 136437098 | 98.1 | C | 0.50 | 0.44 | 0.60 | 0.87 | 1.35E-01 | 1.00E+00 | 2.06E-01 | 1.00E+00 | 1.32E-02 | 1.62E-01 | 3.77E-02 | 1.08E-01 | 4.32E-01 | 3.20E-01 | 4.80E-02 | 2.70E-01 | 1.54E-01 |
| rs4832640 | C/T | 2 | 19025771 | 98.9 | C | 0.29 | 0.24 | 0.43 | 0.71 | 3.64E-01 | 2.10E-01 | 6.22E-01 | 1.00E+00 | 1.97E-02 | 1.86E-01 | 6.27E-02 | 1.15E-01 | 4.69E-01 | 3.54E-01 | 4.13E-02 | 2.83E-01 | 1.35E-01 |
| rs6432110 | A/T | 2 | 10688616 | 99.6 | T | 0.46 | 0.43 | 0.39 | 0.26 | 4.58E-01 | 1.00E+00 | 1.28E-01 | 3.10E-01 | 7.81E-04 | 3.89E-02 | 0.00E+00 | 1.62E-02 | 1.70E-01 | 4.73E-02 | 9.90E-03 | 1.31E-01 | 2.31E-02 |
| rs6745653 | C/T | 2 | 97748515 | 99.3 | T | 0.10 | 0.14 | 0.26 | 0.37 | 1.00E+00 | 6.20E-01 | 3.62E-01 | 5.63E-01 | 1.30E-02 | 1.28E-01 | 3.75E-02 | 3.79E-02 | 2.36E-01 | 1.30E-01 | 6.74E-03 | 1.08E-01 | 1.05E-02 |
| rs1879558 | C/T | 3 | 155137053 | 96.7 | C | 0.24 | 0.16 | 0.22 | 0.19 | 3.00E-01 | 6.78E-01 | 2.88E-01 | 6.66E-01 | 3.05E-03 | 6.15E-02 | 0.00E+00 | 7.02E-04 | 2.86E-02 | 0.00E+00 | 8.27E-04 | 3.30E-02 | 0.00E+00 |
| rs2596834 | C/T | 3 | 10795067 | 99.6 | C | 0.54 | 0.5 | 0.43 | 0.00 | 1.00E+00 | 8.20E-01 | 1.38E-01 | 1.00E+00 | 2.72E-03 | 7.35E-02 | 0.00E+00 | 2.16E-01 | 5.00E-01 | 4.53E-01 | 1.77E-01 | 4.26E-01 | 3.92E-01 |
| rs4686497 | A/G | 3 | 190124209 | 100.0 | A | 0.19 | 0.14 | 0.32 | 0.96 | 1.00E+00 | 1.00E+00 | 1.00E+00 | 1.00E+00 | 2.53E-02 | 1.87E-01 | 8.32E-02 | 4.03E-01 | 8.22E-01 | 7.99E-01 | 2.52E-01 | 6.35E-01 | 5.92E-01 |
| rs822759 | G/T | 3 | 22948116 | 99.3 | T | 0.39 | 0.34 | 0.51 | 0.45 | 8.47E-01 | 4.48E-01 | 1.00E+00 | 1.03E-01 | 1.52E-02 | 1.72E-01 | 4.56E-02 | 6.36E-03 | 1.10E-01 | 9.76E-03 | 1.92E-03 | 6.19E-02 | 0.00E+00 |
| rs9290675 | C/T | 3 | 180489335 | 98.5 | C | 0.13 | 0.18 | 0.20 | 0.12 | 4.22E-01 | 2.28E-01 | 7.18E-01 | 5.52E-01 | 5.60E-04 | 2.62E-02 | 0.00E+00 | 3.28E-03 | 5.71E-02 | 0.00E+00 | 6.52E-03 | 8.33E-02 | 8.59E-03 |
| rs9861816 | C/G | 3 | 195649797 | 99.6 | C | 0.23 | 0.22 | 0.40 | 0.91 | 4.31E-01 | 5.05E-01 | 3.26E-01 | 3.95E-01 | 1.92E-02 | 1.81E-01 | 6.06E-02 | 2.65E-01 | 6.85E-01 | 6.31E-01 | 1.53E-01 | 5.04E-01 | 4.23E-01 |
| rs10516982 | G/T | 4 | 96963279 | 97.4 | T | 0.33 | 0.34 | 0.53 | 0.84 | 2.07E-01 | 7.45E-02 | 2.24E-01 | 6.19E-01 | 1.76E-02 | 1.86E-01 | 5.47E-02 | 1.32E-01 | 4.92E-01 | 3.81E-01 | 5.60E-02 | 3.07E-01 | 1.78E-01 |
| rs1073321 | C/T | 4 | 118870455 | 98.5 | T | 0.27 | 0.21 | 0.19 | 0.23 | **1.06E-05** | **3.07E-02** | 1.06E-01 | 5.10E-02 | 1.88E-04 | 1.55E-02 | 0.00E+00 | 3.37E-04 | 2.14E-02 | 0.00E+00 | 1.03E-03 | 3.69E-02 | 0.00E+00 |
| rs12502036 | C/T | 4 | 158829193 | 98.5 | C | 0.28 | 0.23 | 0.23 | - | 1.00E+00 | 1.00E+00 | 7.40E-01 | - | 8.16E-07 | 1.08E-03 | 0.00E+00 | - | - | - | - | - | - |
| rs1373557 | G/T | 4 | 167601812 | 97.8 | T | 0.26 | 0.18 | 0.24 | 0.12 | 8.10E-01 | 1.00E+00 | **4.10E-02** | 1.00E+00 | 3.08E-03 | 6.35E-02 | 0.00E+00 | 3.28E-03 | 5.71E-02 | 0.00E+00 | 1.26E-02 | 1.21E-01 | 3.12E-02 |
| rs17443616 | A/G | 4 | 41063093 | 99.6 | A | 0.26 | 0.30 | 0.44 | 0.18 | 8.12E-01 | 5.90E-01 | 5.15E-02 | 6.71E-01 | 1.03E-02 | 1.39E-01 | 2.69E-02 | 1.00E-02 | 1.21E-01 | 2.27E-02 | 4.01E-02 | 2.59E-01 | 1.28E-01 |
| rs1873195 | C/T | 4 | 38713739 | 98.9 | T | 0.32 | 0.28 | 0.26 | 0.07 | 1.00E+00 | 7.78E-01 | 1.00E+00 | 1.00E+00 | 2.06E-04 | 1.80E-02 | 0.00E+00 | 3.88E-02 | 2.06E-01 | 1.14E-01 | 3.36E-02 | 1.88E-01 | 1.00E-01 |
| rs2014303 | A/C | 4 | 10262125 | 99.6 | A | 0.19 | 0.26 | 0.38 | 0.25 | 3.64E-01 | 2.34E-01 | 6.11E-01 | 2.98E-01 | 9.14E-03 | 1.26E-01 | 2.23E-02 | 2.72E-06 | 2.03E-03 | 0.00E+00 | 9.46E-03 | 1.28E-01 | 2.04E-02 |
| rs2251432 | A/G | 4 | 191063741 | 99.3 | A | 0.15 | 0.24 | 0.28 | 0.29 | 1.00E+00 | 7.56E-01 | 7.63E-01 | 1.00E+00 | 9.97E-04 | 3.92E-02 | 0.00E+00 | 1.65E-03 | 5.06E-02 | 0.00E+00 | 8.11E-05 | 1.15E-02 | 0.00E+00 |
| rs4555709 | C/T | 4 | 53867889 | 99.6 | T | 0.09 | 0.06 | 0.19 | 0.25 | 2.55E-01 | 1.00E+00 | 6.95E-01 | **3.46E-02** | 2.11E-02 | 1.33E-01 | 6.68E-02 | 3.75E-02 | 1.92E-01 | 1.27E-01 | 2.52E-03 | 5.88E-02 | 0.00E+00 |
| rs9328764 | A/G | 4 | 2143685 | 98.9 | A | 0.22 | 0.18 | 0.48 | 0.61 | 1.00E+00 | 4.42E-01 | 1.00E+00 | 5.73E-01 | 4.99E-02 | 2.93E-01 | 1.68E-01 | 9.82E-02 | 4.25E-01 | 3.15E-01 | 8.74E-03 | 1.31E-01 | 1.79E-02 |
| rs974020 | C/T | 4 | 117865302 | 98.1 | C | 0.27 | 0.18 | 0.22 | 0.22 | 4.86E-01 | 4.42E-01 | 4.85E-01 | 4.24E-01 | 8.10E-04 | 3.22E-02 | 0.00E+00 | 8.98E-04 | 3.40E-02 | 0.00E+00 | 2.29E-06 | 1.76E-03 | 0.00E+00 |
| rs153595 | G/T | 5 | 115712081 | 99.3 | T | 0.19 | 0.19 | 0.28 | 0.15 | 7.68E-01 | 2.78E-01 | 7.63E-01 | 1.00E+00 | 5.15E-03 | 8.61E-02 | 6.51E-03 | 1.65E-03 | 4.33E-02 | 0.00E+00 | 1.26E-02 | 1.29E-01 | 3.27E-02 |
| rs16891982 | C/G | 5 | 33987450 | 95.9 | C | 0.34 | 0.15 | 0.66 | 1.00 | 1.36E-01 | 1.00E+00 | 2.80E-01 | 1.00E+00 | 1.41E-01 | 5.05E-01 | 4.15E-01 | 4.69E-01 | 8.49E-01 | 8.28E-01 | 1.37E-01 | 3.43E-01 | 3.15E-01 |
| rs33706 | C/G | 5 | 87540920 | 99.6 | G | 0.26 | 0.29 | 0.38 | 0.52 | 6.31E-01 | 1.77E-01 | 8.00E-01 | 3.09E-01 | 3.86E-03 | 8.28E-02 | 1.53E-03 | 2.64E-02 | 2.24E-01 | 8.69E-02 | 1.02E-02 | 1.42E-01 | 2.46E-02 |
| rs3822616 | A/G | 5 | 94828145 | 99.3 | A | 0.21 | 0.18 | 0.34 | 0.51 | 1.00E+00 | 6.94E-01 | 1.02E-01 | 4.18E-01 | 1.65E-02 | 1.58E-01 | 5.04E-02 | 6.18E-02 | 3.29E-01 | 2.09E-01 | 1.50E-02 | 1.71E-01 | 4.26E-02 |
| rs1032143 | A/G | 6 | 155578295 | 99.6 | G | 0.33 | 0.37 | 0.21 | 0.25 | 6.16E-02 | 6.26E-01 | 1.00E+00 | 2.98E-01 | 1.47E-02 | 1.55E-01 | 4.29E-02 | 7.59E-03 | 1.14E-01 | 1.39E-02 | 1.19E-03 | 4.13E-02 | 0.00E+00 |
| rs10484547 | C/G | 6 | 29560753 | 99.6 | C | 0.08 | 0.06 | 0.15 | 0.30 | 5.01E-01 | 1.00E+00 | 1.00E+00 | 2.26E-01 | 1.07E-02 | 8.78E-02 | 2.82E-02 | 5.18E-02 | 2.37E-01 | 1.75E-01 | 1.65E-02 | 1.50E-01 | 4.88E-02 |
| rs2171209 | C/T | 6 | 159153971 | 98.5 | T | 0.26 | 0.23 | 0.32 | 0.06 | 3.32E-01 | **2.25E-02** | 7.81E-01 | 1.00E+00 | 5.03E-03 | 8.98E-02 | 6.21E-03 | 3.01E-02 | 1.70E-01 | 8.65E-02 | 5.82E-02 | 2.60E-01 | 1.73E-01 |
| rs2187684 | C/T | 6 | 32872697 | 100.0 | C | 0.30 | 0.38 | 0.33 | 0.38 | 8.29E-01 | 6.29E-01 | 1.74E-01 | 5.83E-01 | 1.14E-03 | 4.57E-02 | 0.00E+00 | 1.40E-06 | 1.62E-03 | 0.00E+00 | 1.07E-03 | 4.41E-02 | 0.00E+00 |
| rs2596501 | C/T | 6 | 31429190 | 98.1 | C | 0.38 | 0.36 | 0.36 | 0.44 | 5.55E-01 | 8.05E-01 | 2.85E-01 | 2.72E-01 | 0.00E+00 | 0.00E+00 | 0.00E+00 | 2.76E-03 | 7.27E-02 | 0.00E+00 | 2.76E-03 | 7.27E-02 | 0.00E+00 |
| rs2804756 | C/T | 6 | 698586 | 99.6 | T | 0.10 | 0.08 | 0.10 | 0.10 | 3.33E-01 | 1.00E+00 | 9.89E-02 | 1.00E+00 | 4.93E-04 | 1.77E-02 | 0.00E+00 | 7.54E-04 | 2.21E-02 | 0.00E+00 | 2.76E-05 | 4.41E-03 | 0.00E+00 |
| rs756147 | A/G | 6 | 141672412 | 99.6 | A | 0.05 | 0.06 | 0.12 | 0.16 | 2.17E-01 | 2.68E-01 | 5.83E-01 | 3.25E-01 | 4.24E-03 | 5.27E-02 | 3.07E-03 | 1.24E-02 | 9.87E-02 | 3.50E-02 | 2.20E-03 | 4.60E-02 | 0.00E+00 |
| rs10486207 | A/G | 7 | 7884020 | 99.6 | G | 0.29 | 0.28 | 0.35 | 0.06 | 6.63E-01 | 1.00E+00 | 1.00E+00 | 1.00E+00 | 2.80E-03 | 6.93E-02 | 0.00E+00 | 4.27E-02 | 2.13E-01 | 1.25E-01 | 6.60E-02 | 2.82E-01 | 1.94E-01 |
| rs17864053 | A/G | 7 | 89801957 | 98.5 | A | 0.16 | 0.20 | 0.25 | 1.00 | 7.82E-02 | 7.22E-01 | 5.18E-01 | 1.00E+00 | 1.29E-03 | 4.23E-02 | 0.00E+00 | 4.19E-01 | 7.96E-01 | 7.74E-01 | 3.83E-01 | 7.54E-01 | 7.40E-01 |
| rs1922086 | C/T | 7 | 155515702 | 96.7 | T | 0.46 | 0.34 | 0.58 | 0.68 | 1.00E+00 | 8.02E-01 | 1.00E+00 | 1.00E+00 | 2.88E-02 | 2.38E-01 | 9.57E-02 | 6.05E-02 | 3.44E-01 | 1.99E-01 | 6.04E-03 | 1.06E-01 | 7.14E-03 |
| rs2097884 | A/C | 7 | 4125658 | 99.3 | C | 0.27 | 0.31 | 0.39 | 0.18 | 6.41E-01 | 7.92E-01 | 6.14E-01 | 3.53E-01 | 3.35E-03 | 7.80E-02 | 0.00E+00 | 1.14E-02 | 1.30E-01 | 2.80E-02 | 2.69E-02 | 2.08E-01 | 8.33E-02 |
| rs2219248 | C/T | 7 | 113965977 | 99.6 | C | 0.28 | 0.39 | 0.22 | - | 3.68E-01 | 1.00E+00 | 7.22E-01 | - | 1.70E-02 | 1.69E-01 | 5.14E-02 | - | - | - | - | - | - |
| rs2367191 | A/G | 7 | 141571268 | 98.1 | A | 0.41 | 0.47 | 0.19 | 0.10 | 7.03E-01 | 1.00E+00 | 1.00E+00 | 4.32E-01 | 4.59E-02 | 2.81E-01 | 1.50E-01 | 8.72E-02 | 3.66E-01 | 2.55E-01 | 7.36E-03 | 8.47E-02 | 1.14E-02 |
| rs2905347 | A/G | 7 | 22393559 | 99.6 | A | 0.48 | 0.38 | 0.60 | 0.69 | 7.15E-01 | 6.33E-01 | 4.56E-01 | 1.00E+00 | 2.47E-02 | 2.21E-01 | 8.07E-02 | 4.89E-02 | 3.09E-01 | 1.61E-01 | 4.24E-03 | 8.80E-02 | 2.62E-04 |
| rs10504924 | C/T | 8 | 94157437 | 99.6 | T | 0.10 | 0.12 | 0.12 | 0.00 | 1.00E+00 | 1.00E+00 | 2.15E-01 | 1.00E+00 | 7.05E-07 | 7.64E-04 | 0.00E+00 | 4.23E-02 | 1.17E-01 | 9.19E-02 | 4.26E-02 | 1.18E-01 | 9.73E-02 |
| rs920590 | C/T | 8 | 19695441 | 99.6 | C | 0.45 | 0.32 | 0.48 | 0.45 | 1.40E-01 | 1.91E-01 | 8.11E-01 | 7.87E-01 | 1.23E-02 | 1.53E-01 | 3.45E-02 | 8.90E-03 | 1.30E-01 | 1.99E-02 | 2.75E-04 | 2.34E-02 | 0.00E+00 |
| rs10512122 | C/G | 9 | 81715524 | 96.7 | C | 0.24 | 0.15 | 0.35 | 0.25 | 8.02E-01 | 6.67E-01 | 5.86E-01 | 7.42E-01 | 2.78E-02 | 2.02E-01 | 9.28E-02 | 7.99E-03 | 1.01E-01 | 1.74E-02 | 6.15E-03 | 1.02E-01 | 8.14E-03 |
| rs1408794 | A/G | 9 | 12641340 | 99.6 | A | 0.48 | 0.44 | 0.57 | 0.94 | 7.15E-01 | 8.18E-01 | 8.05E-01 | 1.00E+00 | 8.84E-03 | 1.33E-01 | 2.10E-02 | 1.64E-01 | 5.01E-01 | 4.28E-01 | 1.02E-01 | 3.68E-01 | 2.92E-01 |
| rs2086085 | A/C | 9 | 1680761 | 99.3 | C | 0.15 | 0.14 | 0.16 | 0.13 | 1.00E+00 | 3.44E-01 | 3.43E-01 | 2.63E-01 | 1.40E-04 | 1.20E-02 | 0.00E+00 | 1.36E-04 | 1.14E-02 | 0.00E+00 | 5.52E-04 | 2.34E-02 | 0.00E+00 |
| rs1045873 | A/C | 10 | 25177778 | 95.6 | C | 0.29 | 0.24 | 0.19 | 0.02 | 6.46E-01 | 7.58E-01 | 6.84E-01 | 1.00E+00 | 2.40E-03 | 5.69E-02 | 0.00E+00 | 6.50E-02 | 2.25E-01 | 1.69E-01 | 4.42E-02 | 1.68E-01 | 1.20E-01 |
| rs10508372 | A/G | 10 | 9012024 | 98.9 | A | 0.09 | 0.07 | 0.19 | 0.21 | 5.96E-01 | 1.00E+00 | 6.98E-01 | 1.00E+00 | 1.66E-02 | 1.22E-01 | 5.03E-02 | 2.00E-02 | 1.37E-01 | 6.48E-02 | 1.76E-04 | 1.51E-02 | 0.00E+00 |
| rs10509384 | A/G | 10 | 78693188 | 80.4 | G | 0.20 | 0.26 | 0.12 | 0.04 | 1.41E-01 | 1.00E+00 | 1.94E-01 | 1.00E+00 | 1.58E-02 | 1.38E-01 | 4.48E-02 | 5.37E-02 | 2.20E-01 | 1.49E-01 | 1.25E-02 | 8.27E-02 | 2.86E-02 |
| rs10509954 | A/G | 10 | 113658378 | 98.9 | A | 0.13 | 0.10 | 0.13 | 0.10 | 3.97E-01 | 5.80E-01 | 1.00E+00 | 4.25E-01 | 1.11E-03 | 3.04E-02 | 0.00E+00 | 2.07E-05 | 3.90E-03 | 0.00E+00 | 1.43E-03 | 3.43E-02 | 0.00E+00 |
| rs379773 | C/G | 10 | 109905342 | 96.7 | G | 0.30 | 0.3 | 0.42 | 0.63 | 1.12E-01 | 1.00E+00 | 8.06E-01 | 1.00E+00 | 7.75E-03 | 1.19E-01 | 1.69E-02 | 5.53E-02 | 3.28E-01 | 1.85E-01 | 2.21E-02 | 2.09E-01 | 6.91E-02 |
| rs7908825 | C/G | 10 | 75224547 | 100.0 | C | 0.32 | 0.29 | 0.29 | 0.69 | 5.28E-01 | 5.81E-01 | 1.44E-01 | 7.51E-01 | 2.20E-06 | 1.91E-03 | 0.00E+00 | 8.18E-02 | 3.99E-01 | 2.64E-01 | 8.10E-02 | 3.97E-01 | 2.60E-01 |
| rs1560569 | A/G | 11 | 8896463 | 99.3 | G | 0.34 | 0.33 | 0.20 | 0.15 | 5.42E-01 | 1.00E+00 | 7.14E-01 | 9.90E-02 | 1.10E-02 | 1.30E-01 | 2.90E-02 | 2.11E-02 | 1.74E-01 | 6.21E-02 | 1.67E-03 | 4.40E-02 | 0.00E+00 |
| rs2847502 | C/T | 11 | 119623708 | 97.4 | T | 0.40 | 0.25 | 0.38 | 0.49 | 4.34E-01 | 5.48E-01 | 1.00E+00 | **3.26E-02** | 9.41E-03 | 1.27E-01 | 2.34E-02 | 3.06E-02 | 2.38E-01 | 1.02E-01 | 6.20E-03 | 1.10E-01 | 8.10E-03 |
| rs4938377 | G/T | 11 | 116801502 | 99.6 | G | 0.08 | 0.08 | 0.04 | 0.01 | 5.42E-01 | 1.00E+00 | 1.00E+00 | 1.00E+00 | 4.17E-03 | 4.22E-02 | 1.99E-03 | 1.65E-02 | 6.99E-02 | 3.53E-02 | 4.57E-03 | 2.77E-02 | 0.00E+00 |
| rs7108371 | C/T | 11 | 44509300 | 98.1 | C | 0.50 | 0.38 | 0.61 | 0.51 | 5.76E-01 | 8.09E-01 | 4.39E-01 | 4.46E-01 | 2.80E-02 | 2.35E-01 | 9.23E-02 | 8.82E-03 | 1.32E-01 | 2.02E-02 | 5.46E-03 | 1.04E-01 | 5.88E-03 |
| rs923031 | A/C | 11 | 15777602 | 99.6 | A | 0.42 | 0.44 | 0.34 | 0.12 | 5.70E-01 | 3.61E-01 | 5.87E-01 | 5.52E-01 | 5.62E-03 | 1.03E-01 | 8.40E-03 | 6.80E-02 | 3.23E-01 | 2.06E-01 | 3.55E-02 | 2.20E-01 | 1.10E-01 |
| rs1003306 | A/T | 12 | 2624458 | 99.6 | A | 0.33 | 0.32 | 0.49 | 0.69 | 3.00E-01 | 1.93E-01 | 1.00E+00 | 5.43E-01 | 1.59E-02 | 1.74E-01 | 4.83E-02 | 7.15E-02 | 3.73E-01 | 2.33E-01 | 2.07E-02 | 1.99E-01 | 6.37E-02 |
| rs1582398 | A/G | 12 | 78624019 | 98.5 | G | 0.15 | 0.10 | 0.35 | 0.98 | 1.00E+00 | 1.00E+00 | 1.00E+00 | 1.00E+00 | 4.63E-02 | 2.47E-01 | 1.55E-01 | 4.86E-01 | 8.85E-01 | 8.71E-01 | 2.73E-01 | 6.37E-01 | 6.12E-01 |
| rs3809125 | C/T | 12 | 55130616 | 98.9 | T | 0.32 | 0.37 | 0.31 | 0.06 | 1.00E+00 | 1.38E-01 | 1.00E+00 | 1.00E+00 | 2.53E-03 | 6.74E-02 | 0.00E+00 | 7.62E-02 | 3.10E-01 | 2.18E-01 | 5.23E-02 | 2.42E-01 | 1.56E-01 |
| rs7965049 | A/G | 12 | 7537044 | 98.5 | A | 0.24 | 0.29 | 0.35 | 0.94 | 1.00E+00 | 2.67E-01 | 4.19E-01 | 1.00E+00 | 1.97E-03 | 5.85E-02 | 0.00E+00 | 2.46E-01 | 6.44E-01 | 5.81E-01 | 2.09E-01 | 5.86E-01 | 5.22E-01 |
| rs998401 | A/G | 12 | 53641500 | 99.6 | A | 0.08 | 0.06 | 0.05 | 0.07 | 1.86E-01 | 1.00E+00 | 1.00E+00 | 1.00E+00 | 4.14E-04 | 1.35E-02 | 0.00E+00 | 1.18E-04 | 7.79E-03 | 0.00E+00 | 9.74E-04 | 2.13E-02 | 0.00E+00 |
| rs1854226 | C/T | 13 | 97036243 | 93.3 | T | 0.13 | 0.26 | 0.10 | 0.07 | 6.69E-01 | 2.12E-01 | 1.00E+00 | 2.39E-01 | 2.26E-02 | 1.60E-01 | 6.97E-02 | 3.24E-02 | 1.84E-01 | 9.54E-02 | 9.52E-04 | 2.43E-02 | 0.00E+00 |
| rs7997100 | C/T | 13 | 67988075 | 99.6 | T | 0.37 | 0.38 | 0.39 | 0.30 | 3.36E-01 | 3.39E-01 | 4.45E-01 | 1.00E+00 | 3.48E-05 | 8.13E-03 | 0.00E+00 | 3.71E-03 | 8.16E-02 | 0.00E+00 | 4.46E-03 | 8.97E-02 | 1.12E-03 |
| rs986642 | C/G | 13 | 56944263 | 99.6 | G | 0.23 | 0.29 | 0.11 | 0.28 | 7.13E-02 | 1.00E+00 | 1.71E-01 | 3.40E-01 | 2.65E-02 | 1.82E-01 | 8.33E-02 | 1.82E-04 | 1.72E-02 | 0.00E+00 | 2.24E-02 | 1.65E-01 | 7.07E-02 |
| rs10483853 | A/G | 14 | 72826052 | 98.1 | G | 0.36 | 0.31 | 0.47 | 0.20 | 4.15E-01 | 1.00E+00 | 6.33E-01 | 6.70E-01 | 1.45E-02 | 1.65E-01 | 4.30E-02 | 7.37E-03 | 1.05E-01 | 1.29E-02 | 4.20E-02 | 2.71E-01 | 1.35E-01 |
| rs10519269 | A/C | 15 | 77705433 | 97.8 | C | 0.12 | 0.10 | 0.23 | 0.50 | 1.00E+00 | 1.00E+00 | 7.37E-01 | 7.91E-01 | 1.49E-02 | 1.27E-01 | 4.44E-02 | 9.92E-02 | 3.96E-01 | 3.21E-01 | 3.96E-02 | 2.69E-01 | 1.32E-01 |
| rs1129038 | C/T | 15 | 26030454 | 98.5 | C | 0.68 | 0.62 | 0.95 | - | 8.33E-01 | 3.34E-01 | **5.93E-03** | - | 8.60E-02 | 3.24E-01 | 2.51E-01 | - | - | - | - | - | - |
| rs7163907 | C/T | 15 | 73632152 | 98.9 | C | 0.37 | 0.29 | 0.34 | 0.56 | **4.98E-02** | 5.84E-01 | 1.00E+00 | 1.00E+00 | 1.92E-03 | 5.76E-02 | 0.00E+00 | 4.04E-02 | 2.79E-01 | 1.36E-01 | 2.50E-02 | 2.22E-01 | 7.93E-02 |
| rs1107820 | C/T | 17 | 41491195 | 99.6 | C | 0.26 | 0.25 | 0.16 | 0.00 | 1.00E+00 | 1.00E+00 | 3.58E-01 | 1.00E+00 | 5.99E-03 | 8.82E-02 | 9.53E-03 | 9.56E-02 | 2.50E-01 | 2.18E-01 | 5.96E-02 | 1.62E-01 | 1.40E-01 |
| rs1476162 | A/G | 17 | 8031808 | 99.6 | G | 0.18 | 0.16 | 0.31 | 0.38 | 5.24E-01 | 1.94E-01 | 7.79E-01 | 9.16E-02 | 1.61E-02 | 1.51E-01 | 4.92E-02 | 3.26E-02 | 2.24E-01 | 1.11E-01 | 2.95E-03 | 7.30E-02 | 0.00E+00 |
| rs2003092 | A/G | 17 | 59516509 | 96.3 | G | 0.31 | 0.34 | 0.41 | 0.73 | 5.10E-01 | 4.47E-01 | 6.18E-01 | 1.00E+00 | 2.34E-03 | 6.63E-02 | 0.00E+00 | 7.75E-02 | 3.87E-01 | 2.48E-01 | 5.36E-02 | 3.21E-01 | 1.75E-01 |
| rs959260 | C/T | 17 | 70881017 | 97.4 | C | 0.25 | 0.19 | 0.34 | 0.91 | 8.02E-01 | 4.51E-01 | 5.90E-01 | 1.00E+00 | 1.50E-02 | 1.52E-01 | 4.49E-02 | 2.92E-01 | 7.18E-01 | 6.70E-01 | 1.87E-01 | 5.66E-01 | 4.90E-01 |
| rs2418844 | A/G | 18 | 26037261 | 97.8 | G | 0.49 | 0.46 | 0.41 | 0.59 | 8.53E-01 | 8.17E-01 | 6.18E-01 | 1.00E+00 | 1.28E-03 | 5.01E-02 | 0.00E+00 | 8.56E-03 | 1.31E-01 | 1.92E-02 | 1.65E-02 | 1.81E-01 | 4.86E-02 |
| rs4892082 | A/C | 18 | 69051672 | 99.6 | A | 0.18 | 0.16 | 0.40 | 0.64 | 5.46E-01 | 4.07E-01 | 1.41E-01 | 2.51E-01 | 3.62E-02 | 2.40E-01 | 1.22E-01 | 1.22E-01 | 4.72E-01 | 3.77E-01 | 2.72E-02 | 2.32E-01 | 8.71E-02 |
| rs523776 | C/T | 18 | 7554299 | 99.3 | T | 0.30 | 0.23 | 0.40 | 0.48 | 6.61E-01 | 2.16E-01 | 8.04E-01 | 4.37E-01 | 1.56E-02 | 1.63E-01 | 4.72E-02 | 3.44E-02 | 2.50E-01 | 1.16E-01 | 3.78E-03 | 8.63E-02 | 0.00E+00 |
| rs959763 | G/T | 18 | 56797826 | 99.3 | G | 0.38 | 0.37 | 0.36 | 0.48 | 5.59E-01 | 1.00E+00 | 1.00E+00 | 1.00E+00 | 5.22E-05 | 9.84E-03 | 0.00E+00 | 6.56E-03 | 1.13E-01 | 1.13E-02 | 7.78E-03 | 1.23E-01 | 1.52E-02 |
| rs103294 | C/T | 19 | 59489660 | 98.1 | T | 0.17 | 0.16 | 0.06 | 0.05 | 5.06E-01 | 4.02E-01 | 1.00E+00 | 1.34E-01 | 1.38E-02 | 1.03E-01 | 3.72E-02 | 1.56E-02 | 1.08E-01 | 3.95E-02 | 6.17E-05 | 5.16E-03 | 0.00E+00 |
| rs202546 | C/T | 20 | 1611539 | 99.6 | T | 0.61 | 0.53 | 0.47 | 0.34 | 1.24E-01 | 3.61E-01 | 1.00E+00 | 5.58E-01 | 1.92E-03 | 6.19E-02 | 0.00E+00 | 1.97E-02 | 1.96E-01 | 6.01E-02 | 9.39E-03 | 1.34E-01 | 2.04E-02 |
| rs477627 | A/G | 20 | 47613465 | 98.1 | A | 0.18 | 0.22 | 0.21 | 0.08 | 1.00E+00 | 1.00E+00 | 1.00E+00 | 2.99E-01 | 9.81E-05 | 1.16E-02 | 0.00E+00 | 2.01E-02 | 1.42E-01 | 5.59E-02 | 1.74E-02 | 1.30E-01 | 4.76E-02 |
| rs7277342 | A/C | 21 | 29040845 | 90.0 | A | 0.34 | 0.29 | 0.48 | 0.95 | 1.11E-01 | 5.90E-02 | **1.68E-04** | **3.57E-03** | 1.81E-02 | 1.84E-01 | 5.64E-02 | 2.59E-01 | 6.55E-01 | 5.99E-01 | 1.51E-01 | 4.71E-01 | 4.06E-01 |
| rs969539 | A/G | 22 | 24942341 | 99.3 | A | 0.38 | 0.39 | 0.16 | 0.00 | 8.47E-01 | 8.12E-01 | 1.90E-01 | 1.00E+00 | 3.38E-02 | 2.31E-01 | 1.10E-01 | 1.61E-01 | 3.95E-01 | 3.59E-01 | 6.06E-02 | 1.64E-01 | 1.43E-01 |

^a^According to NCBI Build 35; ^b^Significant *p*-values in bold. CIs, Canary Islanders; IBE, Iberians; NWA, Northwest Africans; YRI, Yoruba Nigerians.
