# Supplementary material for: North African Influences and Potential Bias in Case-Control Association Studies in the Spanish Population
Source: PLoS One. 2011 Mar 30;6(3):e18389. doi: 10.1371/journal.pone.0018389 (PMC3068190; doi:10.1371/journal.pone.0018389)
Supplement: Table S2 — Pairwise population FST genetic distances. (DOC) [file pone.0018389.s004.doc]

**Table S2.** Pairwise population F_ST_ genetic distances.

| **Populations** | **Canary Is.** | **Iberians** | **Northwest Af.** | **Swedish** | **Polish** | **English** | **Italians** | **Spanish^a^** | **Greeks** |
| --- | --- | --- | --- | --- | --- | --- | --- | --- | --- |
| Iberians | 0.0040 |  |  |  |  |  |  |  |  |
| Northwest Af. | 0.0310 | 0.0500 |  |  |  |  |  |  |  |
| Swedish | 0.0480 | 0.0340 | 0.1340 |  |  |  |  |  |  |
| Polish | 0.0300 | 0.0210 | 0.1060 | 0.0050 |  |  |  |  |  |
| English | 0.0290 | 0.0170 | 0.1050 | 0.0040 | 0.0040 |  |  |  |  |
| Italians | 0.0090 | 0.0140 | 0.0320 | 0.0710 | 0.0450 | 0.0480 |  |  |  |
| Spanish^a^ | 0.0060 | 0.0010 | 0.0600 | 0.0300 | 0.0150 | 0.0150 | 0.0160 |  |  |
| Greeks | 0.0100 | 0.0130 | 0.0270 | 0.0720 | 0.0450 | 0.0480 | 0.0020 | 0.0170 |  |
| CEU^b^ | 0.0260 | 0.0150 | 0.0990 | 0.0010 | 0.0020 | 0.0000 | 0.0460 | 0.0110 | 0.0460 |

^a^From Price et al. [30]; ^b^Utah residents with ancestry from northern and western Europe from HapMap [43].
