# Supplementary material for: North African Influences and Potential Bias in Case-Control Association Studies in the Spanish Population
Source: PLoS One. 2011 Mar 30;6(3):e18389. doi: 10.1371/journal.pone.0018389 (PMC3068190; doi:10.1371/journal.pone.0018389)
Supplement: Table S3 — Functional annotation of EuroAIMs within RefSeq genes. (DOC) [file pone.0018389.s005.doc]

| **Table S3** Functional annotation of EuroAIMs within RefSeq genes. | | | | |
| --- | --- | --- | --- | --- |
| rs# | Entrez ID | Gene Symbol | Gene Description | Function Class |
| rs1003306 | 775 | *CAC1C* | Calcium channel, voltage-dependent, L type, alpha 1C subunit | Intron |
| rs1032143 | 26230 | *TIAM2* | T-cell lymphoma invasion and metastasis 2 | Intron |
| rs103294 | - | - | - | - |
| rs1045873 | 56952 | *PRTFDC1* | Phosphoribosyl transferase domain containing 1 | UTR |
| rs10483853 | 8650 | *NUMB* | Numb homolog (Drosophila) | Intron |
| rs10484547 | - | - | - | - |
| rs10486207 | 113263 | *GLCCI1* | Glucocorticoid induced transcript 1 | Intron |
| rs10496610 | - | - | - | - |
| rs10504924 | - | - | - | - |
| rs10508372 | - | - | - | - |
| rs10509384 | 3778 | *KCNMA1* | Potassium large conductance calcium-activated channel, subfamily M, alpha member 1 | Intron |
| rs10509954 | - | - | - | - |
| rs10512122 | - | - | - | - |
| rs10516982 | - | - | - | - |
| rs10519269 | - | - | - | - |
| rs1073321 | - | - | - | - |
| rs1107820 | 284058 | *KIAA1267* | Hypothetical protein LOC284058 | Intron |
| rs1129038 | 8924 | *HERC2* | Hect domain and RLD 2 | UTR (miRNA target site) |
| rs1157492 | 3776 | *KCNK2* | Potassium channel, subfamily K, member 2 | Intron |
| rs11807062 | 63976 | *PRDM16* | PR domain containing 16 | Intron |
| rs12502036 | - | - | - | - |
| rs1364394 | - | - | - | - |
| rs1373557 | - | - | - | - |
| rs1408794 | - | - | - | - |
| rs1416467 | - | - | - | - |
| rs1448314 | - | - | - | - |
| rs1476162 | 54785 | *C17orf59* | Chromosome 17 open reading frame 59 | miRNA target site |
| rs1517407 | - | - | - | - |
| rs153595 | - | - | - | - |
| rs1560569 | 56672 | *C11orf17* | Chromosome 11 open reading frame 17 | Intron |
| rs1582398 | - | - | - | - |
| rs16891982 | 51151 | *SLC45A2* | Membrane associated transporter | Coding (F373L) |
| rs17443616 | - | - | - | - |
| rs17864053 | - | - | - | - |
| rs1854226 | - | - | - | - |
| rs1873195 | 92689 | *FAM114A1* | Family with sequence similarity 114, member A1 | Intron |
| rs1879558 | - | - | - | - |
| rs1890131 | - | - | - | - |
| rs1922086 | - | - | - | - |
| rs2003092 | 2081 | *ERN1* | Endoplasmic reticulum to nucleus siglling 1 | Intron |
| rs2014303 | 116449 | *MIST* | Mast cell immunoreceptor sigl transducer | Intron |
| rs202546 | - | - | - | - |
| rs2086085 | - | - | - | - |
| rs2097884 | - | - | - | - |
| rs2171209 | 94120 | *SYTL3* | Syptotagmin-like 3 | Intron |
| rs2187684 | - | - | - | - |
| rs2219248 | - | - | - | - |
| rs2236876 | 8995 | *TNFSF18* | Tumor necrosis factor (ligand) superfamily, member 18 | Intron |
| rs2251432 | - | - | - | - |
| rs2367191 | - | - | - | - |
| rs2418844 | - | - | - | - |
| rs2419063 | 339479 | *FAM5C* | Family with sequence similarity 5, member C | Intron |
| rs2596501 | - | - | - | - |
| rs2596834 | - | - | - | - |
| rs2804756 | - | - | - | - |
| rs2847502 | 25833 | *POU2F3* | POU domain, class 2, transcription factor 3 | Intron |
| rs2905347 |  | - | - | - |
| rs33706 | 153396 | *TMEM161B* | Transmembrane protein 161B | Intron |
| rs3769005 | 4175 | *MCM6* | MCM6 minichromosome maintence deficient 6 (MIS5 homolog, S. pombe) (S. cerevisiae) | Intron |
| rs379773 | - | - | - | - |
| rs3809125 | 4284 | *MIP* | Major intrinsic protein of lens fiber | UTR |
| rs3822616 | 9652 | *KIAA0372* | KIAA0372 | Intron |
| rs4555709 | 152579 | *SCFD2* | Sec1 family domain containing 2 | Intron |
| rs4686497 | - | - | - | - |
| rs477627 | 5740 | *PTGIS* | Prostaglandin I2 (prostacyclin) synthase | Intron |
| rs4832640 | - | - | - | - |
| rs4892082 | 400655 | *LOC400655* | Hypothetical gene LOC400655 | Intron |
| rs4938377 | - | - | - | - |
| rs495347 | - | - | - | - |
| rs523776 | - | - | - | - |
| rs6432110 | 79954 | *NOL10* | Nucleolar protein 10 | Intron |
| rs6745653 | - | - | - | - |
| rs7108371 | - | - | - | - |
| rs7163907 | 5780 | *PTPN9* | Protein tyrosine phosphatase, non-receptor type 9 | Intron |
| rs725974 | - | - | - | - |
| rs7277342 | - | - | - | - |
| rs7552548 | - | - | - | - |
| rs756147 | - | - | - | - |
| rs7908825 | 23053 | *KIAA0913* | KIAA0913 | Intron |
| rs7965049 | 9332 | *CD163* | CD163 antigen | Intron |
| rs7997100 | - | - | - | - |
| rs822759 | - | - | - | - |
| rs920590 | - | - | - | - |
| rs923031 | - | - | - | - |
| rs9290675 | - | - | - | - |
| rs9328764 | 353497 | *POLN* | Polymerase (D directed) nu | Coding (C424R) |
| rs959260 | 2885 | *GRB2* | Growth factor receptor-bound protein 2 | Intron |
| rs959763 | - | - | - | - |
| rs969539 | 23544 | *SEZ6L* | Seizure related 6 homolog (mouse)-like | Intron |
| rs974020 | - | - | - | - |
| rs9861816 | 79572 | *ATP13A3* | ATPase type 13A3 | Intron |
| rs986642 | - | - | - | - |
| rs998401 | 9840 | *KIAA0748* | KIAA0748 gene product | Intron |
