# Supplementary material for: North African Influences and Potential Bias in Case-Control Association Studies in the Spanish Population
Source: PLoS One. 2011 Mar 30;6(3):e18389. doi: 10.1371/journal.pone.0018389 (PMC3068190; doi:10.1371/journal.pone.0018389)
Supplement: Text S1 — Ancestry informativeness of EuroAIMs in samples from HGDP. (DOC) [file pone.0018389.s006.doc]

**Ancestry informativeness of EuroAIMs in samples from HGDP**

The initial evidence of ancestry informativeness of EuroAIMs for African and Spanish populations was obtained by comparing data from Mozabite Algerians and French Basques, the closest population proxy to Spanish included in HGDP, obtained using a commercial genome-wide SNP genotyping array [32]. We assumed that EuroAIMs were able to differentiate Algerians and Basques from sub-Saharan Africans given the sharp genetic differences of the latter population [32]. In the comparison of Mozabite Algerians and French Basques, the average F_ST_ for the 44 EuroAIMs included in the array was in the 85^th^ percentile of the distribution for the ~642.000 markers genotyped (F_ST_=0.0669), which is in the range of values allowing to separate NNW and SSE European populations [30]. As expected, this comparison also evidenced the availability of a few thousands AIMs showing large allele frequency differences (>0.3) that would be potentially useful to enhance the detection of genetic ancestries for these two populations.
