# Supplementary material for: North African Influences and Potential Bias in Case-Control Association Studies in the Spanish Population
Source: PLoS One. 2011 Mar 30;6(3):e18389. doi: 10.1371/journal.pone.0018389 (PMC3068190; doi:10.1371/journal.pone.0018389)
Supplement: Text S2 — Ancestry informativeness of EuroAIMs subsets. (PDF) [file pone.0018389.s007.pdf]

Ancestry informativeness of EuroAIMs subsets

Given that EuroAIMs were not ascertained to distinguish the population ancestries under study, we reasoned that subsets of markers would perform similarly in identifying populations and measuring African influences, potentially translating into reductions of genotype costs in future studies. Therefore, we explored the ability of subsets of 23, 46 and 69 markers ranking higher for different measures of ancestry informativeness to estimate individual membership to Iberian and Northwest African populations. For all subsets, STRUCTURE correctly identified the two populations and the individual membership to populations was fairly accurate as shown in the figure below:

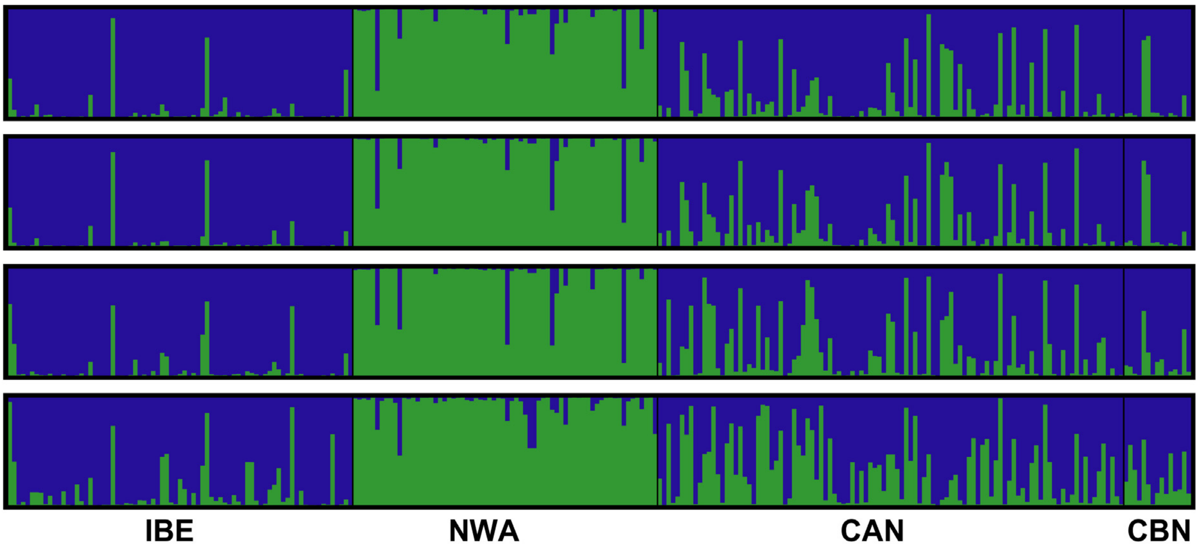

STRUCTURE results based on different subsets of EuroAIMs. Ancestry inference of Canary Islanders collected for this study (CAN), Canary Islanders available from the National Spanish DNA Bank (CBN), Iberians (IBE) and Northwest Africans (NWA) using STRUCTURE based on 93 EuroAIMs and on the subsets of 69, 46 and 23 markers (from top to bottom). Each vertical line represents an individual where colors indicate the proportion of the individual's genome derived from each of the two inferred populations.

As shown in the table below, individual misclassification rate was lowest when 69 EuroAIMs were used and largest when only 23 of these were employed. Significant differences in individual misclassification were found only between runs with 69 and 23 EuroAIMs (4.1 % vs 11.0 %, respectively; two-tailed χ^2 test p=0.026).

Sample misclassification and mean Northwest African influence in Canary Islanders with EuroAIMs subsets.

EuroAIMs subset	F_{ST}[a]	Northwest Africans[b]	Iberians[b]	Northwest Af. influence
93	0.0422	5 (7.4%)	4 (5.2%)	0.168
69	0.0568	3 (4.4%)	3 (3.9%)	0.171
46	0.0805	5 (7.4%)	5 (6.5%)	0.219
23	0.1165	4 (5.9%)	12 (15.6%)	0.333

[a]Average F_{ST} between Northwest Africans and Iberians; [b]Cut-off for membership at 0.70 to self-reported population.

Since the Northwest African influence in Iberians was barely appreciable, we only evaluated these subsets for the appropriate estimation of ancestries in Canary Islanders. Our results showed that the estimated Northwest African influences biased upwards with decreasing number of markers as a consequence of the reduction of information, being almost two-fold for 23 EuroAIMs relative to those for 93 and 69 EuroAIMs (see the table above). In addition, as shown in the figure below, individual ancestry estimates obtained from the 93 markers were weakly correlated with those from 23 and 46, but highly correlated with those from 69 EuroAIMs, 65 of which would fit into two iPLEX™ reactions and still be sufficient for the appropriate estimation of ancestries (r^2=0.845).

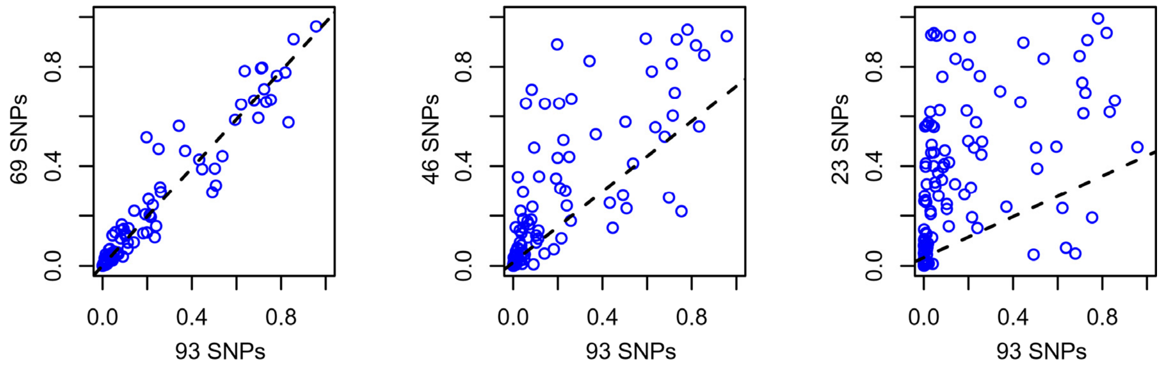

Correlation of estimated Northwest African ancestries in Canary Islanders using subsets of markers. Using those obtained from 93 EuroAIMs as a standard, correlation values (r^2) were 0.930 with those from 69, 0.612 with those from 46, and 0.231 with those from 23.
