# Supplementary material for: North African Influences and Potential Bias in Case-Control Association Studies in the Spanish Population
Source: PLoS One. 2011 Mar 30;6(3):e18389. doi: 10.1371/journal.pone.0018389 (PMC3068190; doi:10.1371/journal.pone.0018389)
Supplement: Text S3 — Details of laboratory procedures. (DOC) [file pone.0018389.s008.doc]

**Details of laboratory procedures**

Genomic DNA from the 104 samples from the Canary Islands was extracted from whole blood using the GFX kit (GE Healthcare, Little Chalfont, UK). All samples used for the study were whole genome amplified from ~10 ng using Illustra GenomiPhi V2 DNA Amplification Kit (GE Healthcare) following manufacturers’ recommendations. In order to verify that the reaction was successful and to quantify the resulting material, a fast SYBR Green I (Molecular Probes, Eugene, OR) real-time-based quantification protocol was established. Briefly, quantifications were performed with a 1/5,000 SYBR Green I final concentration and 1/200 diluted amplified products in 20 μl reactions using iCycler iQ Real-Time detection system (Bio-Rad Laboratories, Hercules, CA), recording the fluorescence at 520 nm under excitation at 490 nm at 25ºC. A DNA concentration standard curve (1000, 750, 250, 100, 10 and 0 ng/ml) was prepared on each plate by including serial dilutions of a DNA molecular weight marker XIV (Roche, Basel, Switzerland). All dilutions were prepared fresh using a 5 mM Tris, 0.5 mM EDTA (pH=7.5) solution. DNA concentrations were obtained from the fluorescence intensities (as relative fluorescence units) interpolated on the standard curve.

Data from nine SNPs that gave poor quality data on the iPLEX™ Gold assay were discarded and finally determined utilizing two alternative simultaneous genotyping reactions (containing 7 and 2 SNPs). Briefly, PCR was performed to amplify two different multiplex reactions, using the HotStartTaq® Master Mix Kit (QIAGEN, Valencia, CA) in reactions of a final volume of 2.5 µl using the Veriti™ 96-Well Fast Thermal Cycler (Applied Biosystems, Foster City, CA) and the following thermal conditions: 94ºC 15 min, followed by 45 cycles of 94ºC 20 sec, 56ºC 30 sec, 72ºC 1 min, and a final extension step of 72ºC 5 min. Reactions were purified by using ExoSAP-IT® (USB Corp., Cleveland, OH) and products used for single base extension reactions using SNaPshot® Multiplex Kit reactions (Applied Biosystems), and fragments resolved on ABI 310 or ABI 3500 (Applied Biosystems).
